# Causes of fetal death in the Flemish cattle herd in Brazil

**DOI:** 10.14202/vetworld.2023.766-772

**Published:** 2023-04-14

**Authors:** Lucas Marian, Jéssica Aline Withoeft, Leonardo da Silva Costa, Luiza Ramos Ribeiro, Isadora Cristina Melo, Raquel Silva Alves, Letícia Ferreira Baumbach, Maicon Gaissler Lorena Pinto, Alessandra Snak, Luiz Claudio Miletti, Sandra Maria Ferraz, Ricardo Antônio Pilegi Sfaciotte, Cláudio Wageck Canal, Renata Assis Casagrande

**Affiliations:** 1 Laboratory of Animal Pathology, Universidade do Estado de Santa Catarina (UDESC), Lages, Santa Catarina, Brazil; 2 Laboratory of Veterinary Virology, Universidade Federal do Rio Grande do Sul (UFRGS) Porto Alegre, Rio Grande do Sul, Brazil; 3 Empresa de Pesquisa Agropecuária e Extensão Rural de Santa Catarina (EPAGRI-SC), Lages, Santa Catarina, Brazil; 4 Laboratory of Parasitology and Parasitic Diseases, UDESC, Lages, Santa Catarina, Brazil; 5 Laboratory of Biochemistry of Hemoparasites and Vectors, UDESC, Lages, Santa Catarina, Brazil; 6 Center for Animal Microbiological Diagnosis, UDESC, Lages, Santa Catarina, Brazil

**Keywords:** abortion, endangered breeds, pathology, protozoan, reproductive disorders

## Abstract

**Background and Aim:**

Flemish cattle in Brazil are on the brink of extinction and are found only in one herd in Lages, Santa Catarina State. This study aimed to uncover the reasons for the recurring abortions in the Flemish cattle herd.

**Materials and Methods:**

Seventeen Flemish fetuses underwent postmortem examinations, with samples collected for histopathology and microbiology culture tests, polymerase chain reaction (PCR) test for *Neospora caninum*, and reverse transcription-PCR (RT-PCR) test for bovine viral diarrhea virus (BVDV) from 2015 to 2020.

**Results:**

Of the 17 fetuses, *N. caninum* was the most common diagnosis and was found in 88% (15/17). One fetus (5.8%) had a coinfection with *N. caninum* and *Citrobacter amalonaticus*, leading to fibrinonecrotic pericarditis. All fetuses tested negative for BVDV by RT-PCR. Of the 107 dams tested by indirect immunofluorescence assay, 26 (25.2%) were anti-*N. caninum* seropositive, with 17 (65.4%) aborting and 5 (19.2%) having estrus repetition. Reverse transcription-PCR results showed that 9 (8.4%) of the serum samples collected from dams tested positive, which tested follow-up test 3 months later, indicating a BVDV transient infection. The factors that contributed to neosporosis included dogs’ access to pastures and improper disposal of fetal remains, which made it easier for dogs to consume them.

**Conclusion:**

This study warns the occurrence of *N. caninum* as a cause of reproductive disorders that can lead to abortion in the studied Flemish cattle herd.

## Introduction

Brazil is a major player in cattle breeding, boasting an estimated herd of 218.2 million animals [1]. Despite its global prominence, the high incidence of fetal mortality due to infectious diseases and dystocia hinders the growth and productivity of the national livestock industry [2–5]. Infections are a significant cause of fetal death in cattle both in Brazil and globally [5, 6]. In Southern Brazil, there is a range of etiologies contributing to high levels of specific agents, particularly *Neospora caninum* [5–9].

Identifying the cause of miscarriages can be challenging due to tissue breakdown and multiple diagnostic techniques are often necessary to confirm if the cause is infectious or non-infectious [2, 5]. The ultimate goal of a proper diagnosis is to implement health controls in herds, ensuring the well-being of animals and people that come into contact with them [10]. In addition to financial losses from decreased reproductive efficiency in commercial herds, the presence of infectious agents can also threaten the survival of endangered breeds such as the Flemish [5, 11–13], which has a European origin and is at risk of extinction. In Brazil, the remaining pure specimens of the breed can only be found in a small herd located in the south of the country [14, 15].

Therefore, this study aimed to detect the causes of abortions through the association of histological, molecular, serological, and epidemiological analyses, identifying potentially infectious agents in the only Flemish herd in Brazil.

## Material and Methods

### Ethical approval

All procedures involving animals used in the study were approved by the Ethics Committee on Animal Use of Animals, under protocol number 9084250817.

### Study period and location

The study examined abortion cases in the Flemish herd in Lages, Santa Catarina, Brazil, from September 2015 to August 2020. The herd was composed of approximately 100 dairy and beef cattle dams, both purebred and crossbred, of various ages.

### Epidemiological assessment of the herd

A technical visit was conducted on the farm for an epidemiological survey. The farm is located within the urban area of Lages, Brazil, and borders residential neighborhoods. The farming system was semi-extensive, with a mix of beef and dairy cattle of the Flemish breed.

The recovery of fetuses and placentas was poor, and in many cases, they remained in a state of decomposition in the environment. Neighborhood dogs frequently visited the farm and had direct contact with the cattle, facilities, and pastures. They were also seen eating fetal and placental remains.

### Reproductive management

All cows were submitted to fixed-time artificial insemination (FTAI). Bulls were used when the cows showed signs of being in heat. After 30–35 days of each FTAI protocol, the cows were checked for pregnancy by rectal palpation and ultrasound and then followed up monthly to monitor fetal viability and diagnose changes in fetal growth.

### Anatomical and pathological examination of aborted fetuses and additional tests

All fetuses recovered were sent fresh to the Laboratory of Animal Pathology (LAPA-CAV/UDESC) in thermal boxes at a refrigeration temperature of approximately 5ºC. Before necropsy, the crown-rump length of fetuses was measured to determine the gestation period at which abortion occurred [16]. When available, the placenta was examined for possible macroscopic lesions. After external analysis, fetuses were stabilized in the left lateral decubitus position. Abdominal, thoracic, and skull cavities were opened for *in situ* analysis of organs. Liver and lung (4.0 cm × 4.0 cm) samples, and placenta when available, were individually stored in sterile packages for bacteriological aerobic culture. Then, about 5 mL of abomasal content was punctured with a disposable syringe and stored in a collection tube. Finally, fragments of about 2 cm × 1 cm were collected from all organs and placed in 10% buffered formalin, stained with hematoxylin and eosin, and routinely processed for histological analysis.

Brain samples were subjected to a polymerase chain reaction (PCR) for *N. caninum* detection. About 5 g of brain tissue was used for manual DNA extraction, using the phenol-chloroform-isoamyl alcohol method [17]. Primers used in PCR were Nc4-Np21plus/Np6plus [18], amplifying a 337-bp product of the Nc5 region. After PCR amplification, samples were subjected to agarose gel electrophoresis (1.5%). *N. caninum* (Nc1 strain) tachyzoites and autoclaved ultrapure water were used as positive and negative controls, respectively.

Spleen and thymus fragments were subjected to reverse transcription-PCR (RT-PCR) for ruminant pestiviruses, including bovine viral diarrhea virus (BVDV), with primers PanPesti F/PanPesti R, which amplify a product of 118 bp from the region 5’ untranslated region of pestiviruses genome [19]. Refrigerated liver, lung, abomasal content, and placenta samples were aerobically cultured in blood and MacConkey agar for *Brucella* spp. isolation.

### Serological and molecular investigations of infectious agents in the herd

All dams in the herd were punctured in the jugular and/or caudal veins for blood collection (10 mL). The samples were stored in tubes without anticoagulants for serum isolation. Then, serum samples were tested for immunoglobulin G (IgG) anti-*N. caninum* by indirect immunofluorescence assay (IFA) [20]. Sera reacting from a 1:100 dilution onwards were considered positive. Finally, a follow-up blood collection was performed on cows that had been aborted.

Reverse transcription-PCR was performed for BVDV using sera from all animals in the herd obtained in the first collection. To avoid misinterpretation of results, collections were made every 3 months. However, new collections were performed in case of positive results.

## Results

### Reproductive management

Reproductive issues were constant in the herd and constituted the main health challenge. Recurrent abortions were reported at all gestational stages, with a higher occurrence at the middle and final thirds, for both multiparous and primiparous cows.

### Anatomopathological evaluation of aborted fetuses and complementary exams

Regarding the gestational stages at which abortions occurred, 5.8% (1/17) corresponded to the first trimester, 5.8% (1/17) to the second, and 88.2% (15/17) to the third, with 47% (8/17) of cases being males and 53% (9/17) females. The placenta was available for analysis in 47% (8/17) of the cases, with histological lesions in two cases. A diagnosis was established in 88% (15/17) of fetuses, with *N. caninum* being the agent mostly related to abortions (Table-1).

**Table-1:** Causes of fetal mortality in a Flemish herd in Brazil. Results of histologic and molecular analyses for *Neospora caninum* and BVDV in cattle fetuses between 2015 and 2020 from the only Flemish herd in Brazil.

Fetus	Gender	Age (months)	Histologic lesions				PCR *N. caninum*	PCR BVDV	Diagnosis

			Brain	Placenta	Skeletal muscle	Heart			
1	Female	9	-	N/A	-	-	Negative	Negative	Dystocia
2	Female	6	-	N/A	-	-	Positive	Negative	Neosporosis
3	Female	9	-	-	-	-	Positive	Negative	Neosporosis
4	Male	8	-	N/A	-	-	Positive	Negative	Neosporosis
5	Male	8	-	N/A	+	-	Positive	Negative	Neosporosis
6	Male	8	-	N/A	+	+	Positive	Negative	Neosporosis
7	Male	8	-	N/A	+	+	Positive	Negative	Neosporosis + C*itrobacter amalonaticus*
8	Female	8	-	-	-	-	Negative	Negative	Inconclusive
9	Female	3	-	+	-	+	Positive	Negative	Neosporosis
10	Female	7	-	N/A	-	-	Positive	Negative	Neosporosis
11	Male	7	-	-	-	+	Positive	Negative	Neosporosis
12	Male	8	-	N/A	-	-	Positive	Negative	Neosporosis
13	Female	9	-	+	-	-	Positive	Negative	Neosporosis + Dystocia
14	Female	9	-	-	-	-	Positive	Negative	Neosporosis
15	Male	8	-	-	-	-	Positive	Negative	Neosporosis + Dystocia
16	Male	9	-	N/A	-	-	Positive	Negative	Neosporosis + Dystocia
17	Female	8	-	-	+	-	Positive	Negative	Neosporosis
Total			0	2	4	4	15	0	

Occurrence of injury: (+) present; (-) absent. N/A=Not available, PCR=Polymerase chain reaction, BVDV=Bovine viral diarrhea virus, *N. caninum: Neospora caninum* [Source: Prepared by the author].

**Figure-1:**
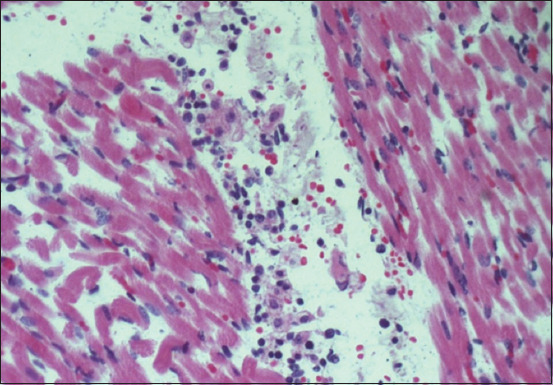
Flemish fetus infected by *Neospora caninum*. Skeletal muscle: Focal moderate lymphoplasmacytic myositis (×40, H and E).

**Figure-2:**
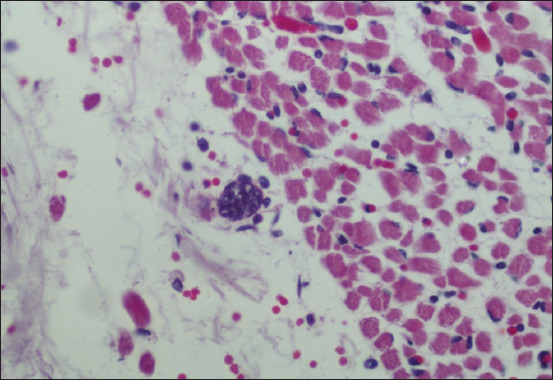
Parasitic structure compatible with *Neospora caninum* cyst containing bradyzoites in Skeletal muscle (×40, H and E).

No macroscopic changes were observed in the organs. All cases were confirmed by PCR in the brain samples. All fetuses showed no histological brain lesions; however, one (5.8%) had moderate multifocal lymphoplasmacytic myositis (Figure-1). It was associated with cyst-like structures, containing bradyzoites, in inflammatory foci (Figure-2). Lesions suggestive of infection by the protozoan were observed in another six cases (35.2%). These were composed of mild multifocal lymphoplasmacytic myositis (66.6%), myocarditis (66.6%), and placentitis (33.3%) isolated or in association. All fetuses evaluated by RT-PCR for BVDV were negative.

**Figure-3:**
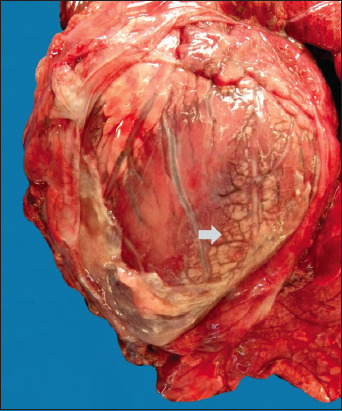
Flemish fetus with concomitant infection of *Neospora caninum* and *Citrobacter amalonaticus*. Heart and pericardial sac: Fibrinonecrotic, multifocal, marked pericarditis. Arrow: Deposition of fibrin filaments in the pericardium.

One *N. caninum*-positive fetus had focally extensive marked fibrinonecrotic pericarditis (Figure-3). Microbiological culture of fetal tissues showed growth of *Citrobacter amalonaticus*, which can be associated with the condition.

One of the non-infectious causes of abortion included fetal dystocia, which was the only cause in one of the cases (5.8%). Dystocia episodes occurred due to late assistance at birth, as no changes in fetal statics were observed. Such complication was also associated with the occurrence of *N. caninum* in three abortion cases (17.6%), which was characterized by marked subcutaneous edema and hemorrhage, predominantly in the cervical region. There were also histological lesions indicative of fetal distress, which were characterized by meconium accumulation in alveoli and bronchioles, in addition to a mild-to-moderate number of multifocal squamous epithelial cells and keratin. This condition was associated with moderate multifocal lymphoplasmacytic infiltrate and characterized as aspiration bronchopneumonia (2/4). Ultimately, only one case (5.8%) had an inconclusive diagnosis.

### Serological and molecular investigations of infectious agents in the herd

**Figure-4:**
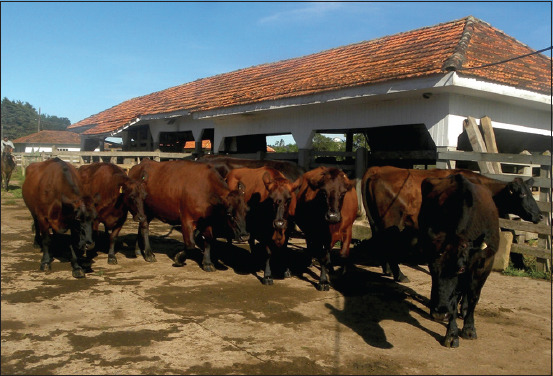
Causes of fetal mortality in a Flemish herd in Brazil. Purebred Flemish dams.

The frequency of anti-*N. caninum* antibodies in the Flemish herd was 25.2% (26/107) (Table-2). Among seropositive females, 10 were crossbred, and 16 were purebred Flemish (Figure-4). As for reproductive age, 50% (13/26) were nulliparous in their first pregnancy, while the remaining 50% (13/26) were multiparous cows of different ages. Increased estrus repetition rates were observed in four seropositive Flemish multiparous cows after four FTAI protocols. Only one out of the four cows with difficulty getting pregnant became pregnant.

**Table-2:** Causes of fetal mortality in a Flemish herd in Brazil. Number of Flemish dams seropositive for *N. caninum* with (n=7) and without (n=19) history of reproductive disorders from a property in the municipality of Lages, SC, Brazil, according to the antibody titer observed in the indirect immunofluorescence reaction (cut-off point≥100).

Reproductive disorder	Antibody title anti-*N. caninum*						

	100	200	400	800	1600	Negative	Total
Abortion and/or return to heat	2	3	0	2	0	11	18
No reproductive disorder	6	8	2	2	1	70	89
Total	8	11	2	4	1	81	107

Source: Prepared by the author, 2022. *N. caninum: Neospora caninum*

Reverse transcription-PCR showed that 8.4% (9/107) of the cows were positive for BVDV in the first analysis. A follow-up blood collection was performed for positive animals 3 months after the first collection, and RT-PCR for BVDV resulted in negative for all animals.

## Discussion

Almost all fetus samples tested showed evidence of infectious agents that can cause abortions in cattle.

However, the exact cause of these abortions in cattle is hardly diagnosed, with only 30 to 40% of the cases being successfully identified among the studies in the literature. This is because many factors contribute to abortion, in addition to tissue damage from autolysis [20]. Nevertheless, this study was more successful in identifying the cause of cattle abortions compared to other studies in the states of Rio Grande do Sul (46.7%) [2] and Santa Catarina (61.17%) [5].

The main cause of cattle abortions was found to be *N. caninum* in 88.2% of the cases, which is consistent with other studies in Brazil and globally [2, 5, 21]. This parasite causes lesions in various parts of the body, including focal necrotizing encephalitis, and lymphoplasmacytic placentitis, myositis, and myocarditis [5, 22, 23]. In this study, seven out of the 15 cases diagnosed with *N. caninum* had characteristic lesions. In one case, multifocal tissue cysts with bradyzoites were observed in the skeletal muscle, where these parasitic structures are most easily found [23].

The cases of *N. caninum*-associated abortion without typical histological lesions were diagnosed by PCR testing of brain samples, which is the organ most affected by neosporosis [24]. This test is highly accurate for detecting *N. caninum* and is considered crucial for diagnosing the condition [5]. Other studies have also found *N. caninum* positivity by PCR testing in fetuses with no histologic lesions, thus, this discrepancy can occur [5, 25]. The uneven distribution of *N. caninum* among tissues can result in typical lesions appearing in some areas while the parasite is found in other regions of the brain [5, 26, 27]. Furthermore, other factors, such as mismanagement or other undiscovered infections, may also play a role in *N. caninum*-related abortions and cannot be fully ruled out.

*Citrobacter amalonaticus* was also found in one fetus using aerobic microbiological culture. The bacteria caused a condition called fibrinonecrotic pericarditis, which is considered to be opportunistic. This type of bacteria has already been reported in fetuses aborted between the middle and late stages of pregnancy [26]. Dystocia was linked to 23.5% of the abortions, either as the only cause or in combination with the parasite *N. caninum*.

This was unexpected because the Flemish cattle are historically known for their ease of calving [13]. The occurrence of dystocia has already been associated with neosporosis [27]. In some cases, *N. caninum* can cause injuries to the heart, brain, and placenta which lead to the early death of the fetus [28]. These injuries may not occur synchronously with full cervical dilation and/or adequate fetal presentation, which are essential for the proper expulsion of the conceptus.

The lesions in the fetuses include visible signs such as hemorrhage in cavities, hemorrhage and edema in the subcutaneous tissue [20], mainly in the head-and-neck area. This may be caused by changes in the fetal position [29]. Aspiration bronchopneumonia is often histologically found along with meconium in alveoli and bronchioles, in addition to squamous epithelial cells and keratin [30]. Meconium can easily clog the airways, leading to lung collapse and pneumonia [31, 32].

A quarter of the herd tested positive for antibodies against *N. caninum* using IFA. This result is similar to the 26.7% positivity reported in the state of Santa Catarina [5] and the 23.1% positivity seen in herds in Lages, Santa Catarina [33]. Detecting *N. caninum* antibodies through serology is crucial for tracking the spread of the disease on farms [10], as seropositive cows were 7.21 times more likely to abort [34]. The serology results from this study, as well as other studies in the state of Santa Catarina, indicate that *N. caninum* is widely spread and shows variations in seropositivity levels, supporting its role as a major cause of reproductive disorders in the Flemish herd.

About 23% (6/26) of *N. caninum* seropositive cows had low conception rates after undergoing sequential FTAI protocols. This was shown by the increase in the number of times they went into heat without getting pregnant. The same problem was observed in other studies, where 72.7% of seropositive cows had trouble getting pregnant despite estrus repetition [5]. The previous research shows that cows with *N. caninum* antibodies need more than two doses of semen to become pregnant [35].

The extensive livestock production on the farm can be associated with the infection because it makes it easier for the animals to ingest sporulated oocysts [36]. Improper disposal of fetal and placental remains, causing them to decompose in the environment, helps keep the disease in the herd [11], which is made worse by the constant presence of stray dogs, which are directly linked to the spread of the disease [36] and hence the cause of abortions [37].

In this herd, 8.4% of the animals tested positive for BVDV using RT-PCR but tested negative in a follow-up test. A previous study showed that 77% of the animals tested were considered to have had a temporary infection after being positive in the first test and negative in the second test [38]. The follow-up test, performed 3 months later, showed that all animals tested negative for BVDV, indicating that the animals were temporarily infected at the time of the first test. This means that there are no permanently infected animals, which are the main cause of virus spread in a herd and result from a fetal infection [39].

Although temporary infections can spread the virus in a herd for a long time through direct contact, it does not require permanently infected animals. The presence of BVDV in the Flemish herd is a warning that permanently infected animals may appear, especially if a female gets infected during the first 3 months of pregnancy [40].

Infectious diseases often compromise the reproductive efficiency of cattle herds of common breeds but are also recurrent in breed groups threatened with extinction in Brazil [41]. The impairment of fertility caused by reproductive disorders can have even more impacting effects on small populations, contributing to the disappearance of breeds with few specimens.

Infections can impair the reproductive ability of cattle herds, even in common breeds, and also frequently happen in endangered breed groups in Brazil [41]. Reproductive problems can have a bigger impact on small populations and lead to the loss of breeds with few members.

## Conclusion

This study highlights the reproductive issues, both infectious and non-infectious, affecting the only Brazilian Flemish herd, putting the breed at risk. Neosporosis is the most common disease and is followed by fetal dystocia. The results of the microbiological analysis suggest that certain opportunistic bacteria may be playing a role in causing fetal loss.

## Authors’ Contributions

RAC, MGLP, LM, JAW, and LSC: Contributed to the planning and development of the study. CWC, LFB, RSA, AS, LCM, SMF, and RAPS: Supplied materials and provided analytical tools. LM, JAW, MGLP, LSC, ICM, and LRR: Conducted experiments with animals. LM, JAW, and RAC: Wrote the manuscript. All authors have read, reviewed, and approved the final manuscript.
